# Reinforcement Learning in Autism Spectrum Disorder

**DOI:** 10.3389/fpsyg.2017.02035

**Published:** 2017-11-21

**Authors:** Manuela Schuetze, Christiane S. Rohr, Deborah Dewey, Adam McCrimmon, Signe Bray

**Affiliations:** ^1^Child and Adolescent Imaging Research Program, University of Calgary, Calgary, AB, Canada; ^2^Behaviour and the Developing Brain, Alberta Children's Hospital Research Institute, University of Calgary, Calgary, AB, Canada; ^3^Department of Neuroscience, University of Calgary, Calgary, AB, Canada; ^4^Hotchkiss Brain Institute, Calgary, AB, Canada; ^5^Department of Radiology, University of Calgary, Calgary, AB, Canada; ^6^Department of Community Health Sciences, University of Calgary, Calgary, AB, Canada; ^7^Department of Pediatrics, University of Calgary, Calgary, AB, Canada; ^8^Educational Psychology, Werklund School of Education, University of Calgary, Calgary, AB, Canada

**Keywords:** reinforcement learning, autism spectrum disorder, conditioning, reward, neuroimaging

## Abstract

Early behavioral interventions are recognized as integral to standard care in autism spectrum disorder (ASD), and often focus on reinforcing desired behaviors (e.g., eye contact) and reducing the presence of atypical behaviors (e.g., echoing others' phrases). However, efficacy of these programs is mixed. Reinforcement learning relies on neurocircuitry that has been reported to be atypical in ASD: prefrontal-sub-cortical circuits, amygdala, brainstem, and cerebellum. Thus, early behavioral interventions rely on neurocircuitry that may function atypically in at least a subset of individuals with ASD. Recent work has investigated physiological, behavioral, and neural responses to reinforcers to uncover differences in motivation and learning in ASD. We will synthesize this work to identify promising avenues for future research that ultimately can be used to enhance the efficacy of early intervention.

## Introduction

Autism spectrum disorder (ASD) is a neurodevelopmental disorder characterized by impairments in communication and social interactions, as well as repetitive and stereotyped behaviors, and restricted interests (American Psychiatric Association, 2013). Although there is no known cure for ASD, early intervention has been shown to improve cognition and adaptive behavior (Dawson et al., 2010; Sullivan et al., 2014) and alter brain responses to social stimuli (Dawson et al., 2012).

Early work showed that operant learning strategies, which involve training a behavior by providing reinforcement, could be used to increase social behaviors such as communication and social interaction (Wolf et al., 1963; Allen et al., 1964; Jensen and Womack, 1967), imitation (Metz, 1965), instruction following (Davison, 1964), and object naming (Martin et al., 1968) in children with ASD. These reinforcement learning (RL) strategies have been refined into a structured and systematic treatment system called Applied Behavioral Analysis (ABA). ABA-based treatment approaches use RL to promote typical social and communication behaviors and to reduce or minimize atypical behaviors (Virués-Ortega, 2010; Dawson and Burner, 2011).

RL-based treatments are supported by evidence and are widely used. Meta-analyses report statistically significant gains with moderate to large effect sizes in treatment groups relative to parent or treatment-as-usual control groups (Remington et al., 2007; Eikeseth, 2009; Dawson et al., 2010; Virués-Ortega, 2010; Reichow et al., 2012). However, it has also been noted that children with ASD vary in the magnitude of their response to ABA-based intervention (Sallows and Graupner, 2005; Sherer and Schreibman, 2005; Schreibman et al., 2009; Perry et al., 2011). Could variable treatment response be related to reduced or inconsistent responses to rewards and/or punishments in ASD, or differences in how the brain forms associations?

Although not a core diagnostic feature in ASD, abnormal responses to rewards and differences in feedback-learning have been documented in several branches of the ASD literature including: social reward (Lin et al., 2012), fear learning (Gaigg and Bowler, 2007), decision-making (Solomon et al., 2015), and perceptual learning (Harris et al., 2015). Although variable response to reinforcement-based therapy is not unique to ASD, this evidence suggests that relying on neurocircuitry associated with operant learning could pose challenges to successful behavior modification in this population. The aim of this non-systematic literature review (Grant and Booth, 2009) is to conduct a survey of the current evidence for atypical RL in ASD in order to address the question of whether abnormal RL, or inter-individual variability, could impact treatment efficacy. The following PubMed search terms were used to retrieve relevant publications: “Autism Spectrum Disorder” AND “Reinforcement Learning,” “Reward,” “Conditioning,” “Intervention,” “Eyeblink Conditioning,” “affective,” “EMG” and a final selection was conducted based on our knowledge from research in the field to include those articles most relevant to the topics under discussion. We also included articles that were cited in these papers covering relevant topics.

We begin by examining whether individuals with ASD respond differently than typically developing (TD) individuals to rewards or punishments Studies on physiological, behavioral, and neural responses to reinforcers in ASD are reviewed. Next, we turn to aversive conditioning studies, which have investigated the formation of passive (Pavlovian) associations between cues and physiological responses. We then review studies of learning and decision making in order to shed light on the learning of stimulus-response-outcome associations. We go on to examine how RL-based treatments change the brain and behavior, and how an individual's response to reinforcers might be consistent with the personal characteristics shown to predict treatment response. Finally, we discuss avenues for future research and implications for improving treatment of ASD symptoms.

## Physiological, behavioral, and neural responses to reinforcers in ASD

Over the course of learning, an association is formed between a cue or action and a reinforcer with some inherent (or previously learned) motivational value. Before considering whether individuals with ASD show abnormalities in learning, a fundamental question that must be considered is whether positive reinforcers (rewards) such as food or social stimuli contain the same motivational value for individuals with ASD as they do for TD individuals. This question is motivated by two theories of ASD. First, the social motivation theory (Chevallier et al., 2012) proposes that individuals with ASD are less motivated to interact with, or orient toward, social stimuli (e.g., faces, eyes, voices), and predicts that typical physiological “reward” responses to social stimuli should be attenuated. Second, the amygdala theory (Baron-Cohen et al., 2000), suggests that impaired function of the amygdala impairs “social intelligence” and understanding of emotional content, and predicts abnormal responses to affective stimuli more generally. Affective stimuli include faces or scenes depicting or invoking specific emotions and may or may not include social content (e.g., both a picture of snakes and a picture of a crying face may be considered negative or unpleasant affective stimuli). Inappropriate physiological responses to either social or affective stimuli could lead to abnormalities in the formation of conditioned associations with these stimuli when they are used as reinforcers. Studies reviewed in this section are detailed in Table 1.

**Table 1:** Participant characteristics of reviewed ASD studies in section Physiological, Behavioral, and Neural Responses to Reinforcers in ASD.

**Author**	**N (Male)**		**DX (Medication details if provided)**	**DX confirmed with**	**Mean Ppt Age (*****SD*****)**		**FSIQ-4 (*****SD*****)**		**VIQ (*****SD*****)**		**PIQ (*****SD*****)**	
	**ASD**	**Control**			**ASD**	**Control**	**ASD**	**Control**	**ASD**	**Control**	**ASD**	**Control**
Anderson et al., 2006	9 (8)	9 (8)	7 AD and 2 PDD-NOS	ADOS-G	4.1	4.1	NA	NA	NA	NA	NA	NA
Blair, 1999	20 (16)	20 (12)	Autism	IC	11.95 (2.81)	6.27 (0.43)	NA	NA	58.8 (11.3)	NA	NA	NA
Cascio et al., 2014	19 (19)	18 (18)	ASD (no psychotropic medication or abstained from stimulants for 24 h)	ADOS and ADI-R	12.58 (2.48)	13.11 (3.44)	109.52 (13.96)	104.22 (12.45)	NA	NA	NA	NA
Cattaneo et al., 2007, Exp 1	7 (6)	8 (4)	HFA	ADOS and ADI-R	6.2	6.5	98	104.6	NA	NA	NA	NA
Cattaneo et al., 2007, Exp 2	8 (7)	8 (4)	HFA	ADOS and ADI-R	6.2	6.5	98	104.7	NA	NA	NA	NA
Damiano et al., 2012	20 (17)	38 (34)	12 HFA, 8 AS	ADOS-G	25.9 (7.96)	20.4 (5.64)	114.7 (11.2)	112.8 (8.6)	112.5 (14.6)	112.9 (9.9)	113.6 (10.3)	109.1 (9.0)
Damiano et al., 2015	24 (23)	21 (17)	ASD (13 with psychotropic medication)	ADOS	14.35 (3.16)	14.25 (2.98)	106.9 (16.3)	110.7 (13.5)	105.7 (17.9)	110.1 (14.2)	106 (15.4)	108.4 (12.0)
Dichter et al., 2010	20 (17)	37 (33)	8 AD, 7 AS, 3 PDD-NOS, 2 not specified (8 with psychotropic medication)	ADI-R	12.4 (1.8)	17.4 (4.8)	NA	NA	NA	NA	NA	NA
Dichter et al., 2012b	16 (14)	20 (14)	2 AS, 14 HFA (9 with psychotropic medication)	ADOS-G	26 (9.1)	25.3 (7)	109.9 (20.3)	127 (8.1)	108.1 (24)	125.6 (9.5)	109.1 (14.1)	122.2 (7.5)
Dubey et al., 2015	30 (21)	24 (14)	ASD	IC or ADOS	20-60	20-60	115.8	118	117.8	117.8	110.1	114.8
Ewing et al., 2013	19 (17)	19 (17)	15 AD, 3 AS, 1 PPD-NOS	ADOS-G	11.3 (2.24)	11.4 (2.7)	NA	NA	100 (12.2)	101.3 (8.8)	98.2 (14.3)	96.8 (7.6)
Hanley et al., 2012^*^	14 (NA)	14 (NA)	AS	ADI-R	20.6	20.4	NA	NA	NA	NA	NA	NA
Hubert et al., 2009	16 (14)	16 (14)	HFA or AS	Autism Spectrum Screening Questionnaire	25.6 (9.2)	27.2 (10.6)	92.2	NA	NA	NA	NA	NA
Joseph et al., 2008	20 (18)	20 (13)	Autism or PDD-NOS	ADOS or ADI-R	12.6 (2.0)	11.11 (1.9)	NA	NA	98 (20)	102 (16)	NA	NA
Klin et al., 2002	15 (15)	15 (15)	AD	ADOS	15.4 (7.2)	17.9 (5.6)	NA	NA	101.3 (24.9)	102.5 (20.4)	NA	NA
Kohls et al., 2012	15 (15)	17 (17)	AS (no psychotropic medication)	ADOS and ADI-R	14.6 (3.3)	13.9 (3.0)	109.8 (12.1)	112.9 (12.6)	NA	NA	NA	NA
Lin et al., 2012	10 (7)	10 (7)	Autism or AS	ADOS and ADI-R	28 (3.1)	27 (3.1)	113 (4.7)	114 (13.4)	NA	NA	NA	NA
Mathersul et al., 2013a	18 (14)	18 (13)	HFA	AQ and/or Ritvo Autism Asperger's Diagnostic Scale	44.6 (15.5)	36.7 (17.1)	117.4	NA	114.4	NA	115.8	NA
Oberman et al., 2009	13 (13)	13 (13)	13 AD, 6 ASD	ADOS-G	10.2 (1.4)	10.2 (1.4)	102.8 (15.8)	112.5 (17.3)	NA	NA	NA	NA
Pierce et al., 2011	37 (30)	51 (16)	27 AD, 9 PDD-NOS, 1 ASD features	ADOS-Toddler Module	2.2 (0.64)	1.89 (0.71)	NA	NA	NA	NA	NA	NA
Riby and Hancock, 2008	20 (15)	20 (14)	Autism	IC	13.4	13.3	NA	NA	NA	NA	NA	NA
Richey et al., 2014	16 (14)	19 (13)	2 AS, 14 HFA (9 with psychotropic medication)	ADOS-G	26 (9.1)	25.3 (7)	NA	NA	NA	NA	NA	NA
Sasson et al., 2012	56 (35)	213 (68)	ASD	AQ	22.5 (5.08)	21.4 (3.1)	NA	NA	NA	NA	NA	NA
Scott-Van Zeeland et al., 2010	16 (16)	16 (16)	6 ASD, 10 Autism (2 with psychostimulants, 2 atypical antipsychotics only, 3 both, 1 SSRI, 1 atypical antidepressant, 1 unknown)	ADOS and ADI-R	12.4 (2.14)	12.3 (1.76)	112.3 (13.6)	119 (8.4)	108.8 (14.9)	119.4 (12.6)	114.1 (13.1)	111.5 (8.1)
Sepeta et al., 2012^*^	20 (NA)	18 (NA)	Autism or AS	ADI-R or ADOS-G	12.4	13.7	106 (20)	117 (12)	101 (20)	117 (13)	110 (18)	112 (11)
Wilbarger et al., 2009	14 (11)	14 (12)	AD or AS	ADI-R or ADOS-G	21.9 (7.5)	21.1 (5.7)	NA	NA	NA	NA	NA	NA

*AD, Autistic Disorder; ADI-R, Autism Diagnostic Interview-Revised; ADOS, Autism Diagnostic Observation Schedule; AS, Asperger's Syndrome; ASD, Autism Spectrum Disorder; AQ, Autism Quotient; DX, Diagnosis; FSIQ-4, Full-scale Intelligence Quotient with 4 subtests; HFA, High functioning Autism; IC, independent clinicians; NA, not available; PDD-NOS, Pervasive Developmental Disorder not otherwise specified; PIQ, Perceptual Intelligence Quotient; SD, Standard Deviation; VIQ, Verbal Intelligence Quotient*.

* *Sepeta et al. (2012) state details for recruited subjects but don't specify which sex their excluded subjects were, Hanley et al. (2012) don't provide gender details*.

### Physiological responses

Different tasks have been used to measure responses to social and affective stimuli in ASD, including passive viewing of static pictures (Wilbarger et al., 2009), viewing of video clips (Hubert et al., 2009), overt ratings of emotional features (Hubert et al., 2009), and ratings of non-emotional features such as gender of faces (Hubert et al., 2009). During these tasks, physiological responses can be quantified by measuring muscular contractions, skin conductance response, or pupil dilation, and can provide insight into affective (“emotional”) responses in ASD.

Muscular contractions in response to startle or facial muscle responses (i.e., smiling and frowning) can be measured with electromyography (EMG). One study of adolescents and young adults with ASD found typical facial muscle and eye blink startle responses to negative pictures from the International Affective Picture System (IAPS), but potentiated startle response to positive IAPS pictures, despite typical facial EMG and overt ratings (Wilbarger et al., 2009). Exaggerated eyeblink responses to pleasant IAPS images were also shown in a study by Dichter et al. (2010), as well as exaggerated postauricular muscular responses to unpleasant images. Neither of these studies provided measures of intellectual functioning for their samples. A third study in adults with ASD who showed no cognitive impairment (often referred to as “high functioning ASD”) found no difference in eyeblink or facial EMG measurements to pleasant and unpleasant IAPS pictures (Mathersul et al., 2013a). These authors note that in contrast to previous studies, the images they used were more consistent, containing only social affective scenes, rather than image sets comprised of social and non-social images, which may have led to a more consistent overall response.

Facial EMG can also be used to measure spontaneous mimicry of others' facial expressions (Hess and Blairy, 2001), and studies in ASD have found some evidence for atypical mimicry (Cattaneo et al., 2007; Oberman et al., 2009). One study recorded mouth-opening mylohyoid (MH) muscle activity in participants while they watched an experimenter grasp and eat food or grasp a piece of paper and place it in a container on his shoulder (Cattaneo et al., 2007). TD children showed increased MH activity during the food grasping compared to the paper-grasping action while MH activity in children with ASD did not differ between conditions. Another study looking at involuntary mimicry recorded EMG from five groups of facial muscles while children categorized emotions of 192 facial expressions shown on a screen (Oberman et al., 2009). These authors found a significant delay of peak facial mimicry in children with ASD compared to TD children—even though the groups did not differ in their ability to categorize the presented emotions. These studies suggest that spontaneous mimicry is altered in children with ASD. A study of high-functioning adults with ASD also found attenuated spontaneous mimicry when images of emotional facial expressions were presented briefly (i.e., backwards masked), independent of whether participants judged emotions or gender of the images (Mathersul et al., 2013b). Since spontaneous mimicry is modulated by the reward value of stimuli (Sims et al., 2012) this could be linked to the reduced motivational value of facial expressions in this population. In contrast, another study that recorded EMG looked at two groups with ASD: one group of children with high functioning ASD and one group of children with ASD with severe impairments of social responsiveness (Social Responsiveness Scale values > 75, Constantino and Gruber, 2012). They found no differences in facial mimicry between high functioning and control children, but children with ASD and severe social impairments showed significantly decreased facial mimicry when watching film clips in which different children expressed anger, sadness, happiness, and fear (Deschamps et al., 2015). In general, EMG based markers show some sensitivity to atypical processing of social and affective information and the latter study hints at variability within the autism spectrum as an underlying cause for atypical mimicry. Future studies should collect additional information on symptom severity in order to fully understand differences in ASD subgroups.

Skin conductance response (SCR) is a sympathetic sweat response that can be evoked by emotional stimuli and is sensitive to arousal (“alertness due to stimuli”), rather than valence (“pleasantness of stimuli”) (Greenwald et al., 1989; Lang et al., 1993). Children with ASD have shown typical SCRs to distress cues (e.g., picture of a crying face) and atypical responses to threatening objects (e.g., picture of a gun; Blair, 1999); both of these types of stimuli are associated with high arousal and negative valence. A study on SCRs during passive viewing of pictures of neutral faces reported increased SCR amplitude in children and adolescents with ASD (Joseph et al., 2008), suggesting increased arousal. However, it is unclear whether increased arousal to facial stimuli is due to eye contact or not: while some studies measuring pupil dilation (Bradley et al., 2008) and SCR (Kylliäinen and Hietanen, 2006) showed increased arousal toward direct eye contact, another study using SCR showed that individuals with ASD did not differ in their SCR based on gaze direction (Joseph et al., 2008). In contrast to these studies in children, adults with ASD showed smaller SCRs relative to TD adults when judging facial emotions from a set of three video clips during which facial expressions evolved from neutral to happy or angry (Hubert et al., 2009). This SCR reduction was interpreted as decreased arousal for emotional faces; however, it was notably specific to an emotion judgment condition and was non-significant when participants judged the age of the same faces. Discrepant findings between studies measuring SCR, that is, hyper- vs. hypo-responses, may point to a developmental trajectory wherein hyper-arousal to faces and direct eye gaze in childhood may attenuate toward hypo-arousal in adulthood. However, differences in the paradigms used across studies (static pictures vs. video-clips, passive viewing vs. emotion-rating) make it difficult to draw firm conclusions. Studies using harmonized task paradigms across a wide age range, and longitudinal work, could help to resolve these questions.

Pupil dilation can measure arousal levels that are non-specific as to valence (Bradley et al., 2008): both pleasant and unpleasant stimuli can evoke dilation during passive viewing of face and non-face stimuli (Anderson et al., 2006; Sepeta et al., 2012). In young children (~4y) with ASD, a pupillary contraction was observed when viewing children's faces that was not seen when viewing landscapes, toys, or animal faces (Anderson et al., 2006). In another study of older children and adolescents, happy faces with gaze directed toward the viewer led to increased pupil diameter in TD but not children with ASD, relative to faces with averted gaze (Sepeta et al., 2012).

Taken together, studies using physiological measures do suggest that there are differences in how social and affective stimuli are processed in individuals with ASD. However, there are inconsistencies as to whether individuals with ASD display hypo- (Hubert et al., 2009; Sepeta et al., 2012) or hyper-arousal (Blair, 1999; Kylliäinen and Hietanen, 2006; Joseph et al., 2008), and the lack of valence information available from physiological techniques makes these results challenging to interpret. Furthermore, large studies that take developmental stage and treatment history into account are lacking, e.g., typical facial muscle and eye blink responses in the studies looking at adult samples could be attributed to successful treatment. Studies including subgroups with different treatment backgrounds, or factoring in the years of treatment, as well as comparing child with adult populations can draw a more detailed picture of symptom trajectories. Nonetheless, this work supports the contention that atypical or variable physiological responses to social and affective stimuli could impact the formation of learned associations between these stimuli and behaviors in individuals with ASD.

### Behavioral responses

While physiological markers such as SCR and pupil diameter suggest differences in emotional processing in individuals with ASD, these measures are non-specific to valence (pleasant or unpleasant), making the interpretation of group differences challenging. A valence-specific measure of how much a human (or animal) values an outcome is how much effort they will exert to obtain it (Hayden et al., 2007). In a “work-to-view” task participants saw pictures of 40 female faces and 20 cars for 800 ms and could either extend the viewing time to up to 5 s by pressing an effortful key combination, or they saw a blank screen until 5 s had passed (Ewing et al., 2013). This task design made it possible for the overall length of the task not to increase when participants “worked to view” a picture. Results showed that children with ASD did not exert less effort to view pictures of faces than a group of TD children (Ewing et al., 2013) and that both groups modulated their effort by the attractiveness of the faces. In contrast, when having to choose to view one of two movie clips that required varying amounts of effort (clips had to be unlocked by pressing a key one, two or three times; Dubey et al., 2015), adults with ASD were less willing to work to view social movies that included direct gaze compared to TD individuals. These effort-based paradigms suggest more typical social “wanting” in children with ASD relative to adults, but given the difference in stimuli (e.g., pictures, movies) it is difficult to draw a firm conclusion. Notably, adults with ASD have also been shown to be differently sensitive to effort expenditure vs. reward, that is, they tend to be more willing to expend effort and are less influenced by changing reward contingencies compared to TD individuals (Damiano et al., 2012), further challenging interpretation of studies using effort-based paradigms.

Overt ratings to assess the subjective value of pictures of people or emotional faces have shown mixed results. While some research has not found a difference between TD and ASD young adults in their rating of subjective pleasantness of emotional facial expressions (Lin et al., 2012), an online study using arousal and valence ratings found significantly lower valence in adults with ASD for “social images” (here, a picture of a single child or adult with a happy expression; Sasson et al., 2012), and higher valence for images related to typical circumscribed interests in ASD (e.g., trains).

Behavioral orienting in the form of attention to social stimuli (eyes, faces, voices) is also reduced in individuals with ASD (Klin et al., 2002; Riby and Hancock, 2008; Hanley et al., 2012). A large online study used the Self-Assessment Manikin (SAM; Bradley and Lang, 1994) to collect valence and arousal ratings on 40 social images (single child or adult with happy expression), 40 High Autism Interest images (HAI; taken from semi-structured parent-report and included trains, electronics, vehicles, construction equipment, airplanes, clocks, blocks, and road signs) and 34 Low Autism Interest images (LAI; clothing, outerwear, office supplies, kitchen supplies, furniture, tools, musical instruments, and plants) (Sasson et al., 2012). They found similar arousal ratings between image categories. However, valence ratings were significantly higher for HAI images compared to social images in the ASD group and the opposite (social images > HAI images) in the TD group (Sasson et al., 2012). Another study showed toddlers videos with 28 scenes of either geometric or social images on monitors next to each other and recorded their eye gaze allocation between monitors as a means of assessing preference (Pierce et al., 2011). They found that toddlers with ASD spent significantly more time on geometric scenes compared to social scenes (Pierce et al., 2011). Studies of visual attention in TD individuals have demonstrated a link between stimulus value and attentional capture (see reviews: Awh et al., 2012; Anderson, 2013), strengthening the argument that differences in allocation of attention in ASD may be related to differences in the relative value of social stimuli.

In summary, effort-based behavioral tasks have an advantage over physiological measures in that they provide valence-specific information. However, an underlying assumption of such tasks is that participants with ASD will exert a similar effort to TD controls for a given reward, which may not be appropriate (Damiano et al., 2012). Nonetheless, the demonstration that individuals with ASD are less willing to work to view social movies with direct gaze (Dubey et al., 2015) suggests reduced motivational value of specific social stimuli. This finding is supported by studies showing lower subjective ratings (Sasson et al., 2012) and reduced attention to social stimuli (Pierce et al., 2011), all of which could indicate that individuals with ASD are less likely to learn when social or socially mediated rewards are used.

### Neural responses

A network of brain regions is sensitive to the hedonic value of stimuli (Bartra et al., 2013). Directly measuring engagement of these regions when an individual is presented with a reward can provide insight into sensitivity to reinforcement. However, this approach should ideally be applied in cooperation with physiological or behavioral measures in order to avoid over-interpreting functional neuroimaging data (Poldrack, 2006).

The ventral striatum (VS), including the nucleus accumbens, receives dopaminergic projections from the ventral tegmental area of the midbrain and plays an important role in reward anticipation (Knutson et al., 2000) and processing of error-feedback during learning (O'Doherty et al., 2003), including social learning (Bray and O'Doherty, 2007). Diminished VS activation in an ASD sample (compared with a TD control sample) during social reward anticipation may indicate diminished social “wanting” (Kohls et al., 2012) and lend support to social motivation theories of ASD (Chevallier et al., 2012).

Several functional neuroimaging studies have used incentive delay tasks (Knutson et al., 2000) during which participants are provided with an incentive for making a speeded response to a target. This task allows investigating neural activity during both the anticipation of a reward and the time a reward is given. One study noted reduced activation in the VS in adults with ASD compared to TD individuals during anticipation of social rewards (pictures of faces; Richey et al., 2014), however, diminished social anticipatory response in the VS has not been consistently reported (Dichter et al., 2012b). Other studies have similarly shown reduced activation in the VS during anticipation of monetary rewards in children (Scott-Van Zeeland et al., 2010) and adults (Dichter et al., 2012b) with ASD compared to TD individuals. While several studies have also noted differences in VS response at the time a reward was given, results have differed in whether response was reduced to social rewards only (Scott-Van Zeeland et al., 2010) or to both social and monetary rewards (Kohls et al., 2013) in ASD. Between these two studies (Scott-Van Zeeland et al., 2010; Kohls et al., 2013) the samples differed only in regards to their medication status with some participants taking psychostimulants, atypical antipsychotics or both in the study by Scott-Van Zeeland et al. (2010) (Table 1). These medications are known to affect dopamine transmission (Seeman, 2002) which is also centrally involved in reward learning and could influence study results. Compared to the studies above, another study used the monetary incentive delay task but only provided negative facial expressions and monetary loss and found hypoactivation in both the VS and caudate in individuals with ASD during anticipation of negative faces (Damiano et al., 2015), whereas only the caudate showed hypoactivation in anticipation of monetary loss. The balance of evidence to date suggests abnormal VS engagement in ASD in response to both appetitive and aversive reinforcers that is not specific to social rewards. These abnormalities may represent a neurobiological marker for diminished incentive salience of rewards in ASD (Kohls et al., 2012).

The ventromedial prefrontal cortex (vmPFC) has been implicated in the representation of stimulus value (Bartra et al., 2013) at the time of outcome (O'Doherty et al., 2004), when evaluating options to make a decision (Kable and Glimcher, 2009) and also during reward imagery (Bray et al., 2010). One study modified the monetary incentive delay task by adding one condition that showed objects which were identified as interesting via semi-structured interviews and eye tracking by the ASD group prior to the task (Dichter et al., 2012a). They found enhanced vmPFC activation at the time of outcome for monetary receipt as well as autism-specific objects of interest in adults with ASD relative to TD controls (Dichter et al., 2012a). A study of children with and without ASD used an incentive go/no-go task during which participants had to either press a button (after seeing a downward arrow) or withhold a button press (upward arrow) and were either rewarded (positive facial expression vs. money gain) or punished (neutral facial expression vs. money loss) depending on task performance (Kohls et al., 2013). Similarly, they found enhanced vmPFC activation at the time children with ASD saw a monetary reward outcome (Kohls et al., 2013). Increased vmPFC (Dichter et al., 2012a) and anterior cingulate (Cascio et al., 2014) activation have also been found in response to autism-specific objects of interest in adults with ASD, relative to TD controls. VmPFC responses to social rewards has been mixed: one study found diminished vmPFC activation for both social and monetary reward feedback in individuals with ASD (Kohls et al., 2013) while two other studies (Scott-Van Zeeland et al., 2010; Dichter et al., 2012b) did not observe a difference in vmPFC for social rewards.

The amygdala plays a role in reward and affective salience (Wassum and Izquierdo, 2015). Increased amygdala activation during social reward anticipation has been shown in adults with ASD and this activation correlated positively with social interaction deficits (Dichter et al., 2012b). In contrast, decreased amygdala activation under social reward conditions has been observed in children with ASD (Kohls et al., 2013). These studies used very similar experimental task designs with comparable reward contingencies (Dichter et al., 2012b; Damiano et al., 2015), suggesting that differential findings in children and adults with ASD might be due to an abnormal developmental trajectory in amygdala reactivity to social incentives (Stanfield et al., 2008; Tottenham and Sheridan, 2009; Schumann et al., 2011).

In general, neuroimaging studies have separately examined response to anticipation and receipt of social, monetary, and “circumscribed interest” rewards. This work has shown attenuated neural response during anticipation of both social and monetary rewards (Scott-Van Zeeland et al., 2010; Dichter et al., 2012a; Richey et al., 2014), but typical or enhanced response at the time of outcome (Dichter et al., 2012b; Kohls et al., 2013; Wassum and Izquierdo, 2015), perhaps indicating a disconnection between the desire to obtain a reward and its hedonic effects. The implications of these findings for reinforcement-based treatment could be that although a reward may be liked or preferred by an individual, it may not consistently provoke the expected anticipatory response, which could affect how associations with this “reward” stimulus are learned. Finally, neuroimaging studies should control for medication status in order to understand whether learning abnormalities are due to ASD symptoms or a possible side effect of medication.

### Summary

Physiological, behavioral and neuroimaging measures have each been used to determine whether responses to social and affective stimuli are atypical in ASD. Although there are many inconsistencies in this literature, atypical responses to rewards have been reported in each of these domains. One aspect that remains relatively unexplored, however, is variability between individuals. As the motivational value of rewards is critical to the formation of learned associations, there exists a need to better characterize inter-individual variability in response to social and non-social rewards, as well as developmental trajectories. Notably, to be relevant for treatments starting in early childhood, more research in young children is clearly needed.

## Learning in the context of aversive conditioning

While teaching with rewards rather than punishment is emphasized in contemporary behavioral interventions (Schreibman et al., 2015), studies of learning in an aversive context may nonetheless provide insight into atypical associative learning mechanisms. These studies also provide insight into the formation of Pavlovian associations (where a neutral stimulus comes to elicit a conditioned response) in ASD. Studies reviewed in this section are detailed in Table 2.

**Table 2:** Participant characteristics of reviewed ASD studies in section Learning in the Context of Aversive Conditioning.

**Author**	**N (Male)**		**DX (Medication details if provided)**	**DX confirmed with**	**Mean Ppt Age (*****SD*****)**		**FSIQ-4 (*****SD*****)**		**VIQ (*****SD*****)**		**PIQ (*****SD*****)**	
	**ASD**	**Control**			**ASD**	**Control**	**ASD**	**Control**	**ASD**	**Control**	**ASD**	**Control**
Bernier et al., 2005	14 (12)	14 (12)	AS, Autism or PDD-NOS	ADOS and ADI-R	18.4 (5.5)	19.7 (8.2)	106.1 (13.0)	114.9 (12.5)	NA	NA	NA	NA
Gaigg and Bowler, 2007	14 (12)	14 (11)	AS (none)	IC	29.7 (10.2)	30.4 (12.2)	111 (17.3)	109 (12.7)	NA	NA	NA	NA
Salmond et al., 2003	14 (13)	18 (6)	HFA or AS (none)	IC	12.9	12.6	NA	NA	85-115	85-115	NA	NA
Sears et al., 1994^*^	11 (NA)	11 (NA)	Autism	IC	12.2	12.7	NA	NA	NA	NA	105.7	115.4
South et al., 2011	36 (33)	36 (32)	ASD	ADOS-G	12.38 (2.69)	13.18 (3.05)	105.93 (11.89)	109.43 (9.04)	103.2 (13.90)	108.4 (11.54)	107.23 (12.77)	108.13 (8.32)

*AD, Autistic Disorder; ADI-R, Autism Diagnostic Interview-Revised; ADOS, Autism Diagnostic Observation Schedule; AS, Asperger's Syndrome; ASD, Autism Spectrum Disorder; AQ, Autism Quotient; DX, Diagnosis; FSIQ-4, Full-scale Intelligence Quotient with 4 subtests; HFA, High functioning Autism; IC, independent clinicians; NA, not available; PDD-NOS, Pervasive Developmental Disorder not otherwise specified; PIQ, Perceptual Intelligence Quotient; SD, Standard Deviation; VIQ, Verbal Intelligence Quotient*.

* *Sears et al. (1994) don't provide gender details*.

### Fear conditioning

In fear conditioning paradigms, neutral stimuli come to elicit a physiological response after repeated pairings with aversive stimuli, such as a mild electric shock or loud noise (Ohman, 2009). Both animal and human lesion evidence points to the amygdala and medial temporal lobes as key structures in the acquisition and expression of conditioned fear (LaBar et al., 1995; Morris et al., 1999; LeDoux, 2000; Phelps and LeDoux, 2005).

Learning a simple cue-outcome association leads to a potentiation of the startle response in ASD that is comparable to TD controls (Salmond et al., 2003; Bernier et al., 2005; South et al., 2011) suggesting appropriate fear conditioning in individuals with ASD. A study involving a more complex discriminative learning task with probabilistic feedback found that although fear responses were acquired in both participants with ASD and IQ-matched TD participants, acquired fear responses were weaker and less discriminative in the ASD group (Gaigg and Bowler, 2007).

### Eyeblink conditioning and the cerebellum

In classical eyeblink conditioning, an air puff directed at the face (or mild electric shock near the eyes) is repeatedly paired with a cue such as a light or auditory tone, eventually provoking an eyeblink in response to the cue (Schade Powers et al., 2010). This learning relies on the cerebellum acting in concert with brainstem, hippocampal, and striatal regions (Steinmetz, 2000; Gerwig et al., 2007; Cheng et al., 2008, 2014; Thürling et al., 2015), many of which have been suggested to function atypically in ASD (Jou et al., 2009; Wang et al., 2014).

Eyeblink conditioning has been demonstrated in human infants as young as 1 month (Reeb-Sutherland et al., 2011), making children's responses in this paradigm interesting as early markers for neurodevelopmental disorders (Reeb-Sutherland and Fox, 2015), particularly given the suggested link between early cerebellar injury and ASD (Bolduc et al., 2012; Limperopoulos et al., 2014). In children and young adults with ASD, the eyeblink response is learned faster than in TD controls (Sears et al., 1994) and blink timing occurs earlier than in TD individuals once the response is learned. These authors also noted a trend wherein learning was specifically faster for younger children with ASD relative to TD controls. Further study has shown that this behavior may be particular to delay conditioning, in which the stimulus and outcome temporally overlap (Oristaglio et al., 2013). Social outcomes appear to be learned faster than non-social outcomes in 1-month-old TD infants, indicating an early predisposition toward the salience of social cues (Reeb-Sutherland et al., 2011). This is of particular relevance to ASD as deficits in social communication and interactions are a core symptom. Faster learning at 1 month also correlated with emerging social abilities at 9 months of age (Reeb-Sutherland et al., 2012). While these findings are interesting, the positive correlation between faster eyeblink conditioning and social skills in TD infants (Reeb-Sutherland et al., 2012) is opposite to the pattern seen in older children where learning is faster in ASD children (Sears et al., 1994). As such, the potential for eyeblink conditioning as a biomarker for ASD requires further study in a developmental context and should take treatment history into account.

### Summary

Aversive conditioning paradigms dependent on amygdalar and cerebellar systems have shown that these associations can be learned in individuals with ASD, sometimes more quickly than in TD individuals (Sears et al., 1994). Fear conditioning studies further suggest intact amygdala reactivity, despite reports of aberrant structure and connectivity (Bauman and Kemper, 1985). Yet, as performance was impaired for a more complex fear learning task, this suggests that differences in fear conditioning may be mediated by an impaired fronto-sub-cortical network rather than amygdala dysfunction *per se* (Jarrell et al., 1987; Morris et al., 1997). Although these aversive conditioning paradigms are less representative of learning in an “early intervention” context, this literature can nonetheless be informative in a translational context. Specifically, this work suggests that while fearful or unpleasant stimuli are appropriately processed, learning is more efficacious in situations where task contingencies are straightforward.

## Learning and decision-making

ABA-style therapies rely on operant learning, which can be measured in the laboratory with decision-making tasks: if participants' actions reliably predict a valued outcome, the frequency with which a subject chooses to perform that action provides an index of the strength of the learned association. Studies reviewed in this section are detailed in Table 3.

**Table 3:** Participant characteristics of reviewed ASD studies Learning and Decision-Making.

**Author**	**N (Male)**		**DX (Medication details if provided)**	**DX confirmed with**	**Mean Ppt Age (SD)**		**FSIQ-4 (*****SD*****)**		**VIQ (*****SD*****)**		**PIQ (*****SD*****)**	
	**ASD**	**Control**			**ASD**	**Control**	**ASD**	**Control**	**ASD**	**Control**	**ASD**	**Control**
D'Cruz et al., 2013	41 (33)	37 (31)	22 AD, 12 PDD-NOS, 7 AS (either medication naïve or free from medication for 5.5 half-lives of the drug)	ADOS and ADI-R	15.34 (7.75)	18.24 (8.12)	103.9 (16.48)	108.7 (11.97)	102 (18.7)	109 (12.59)	104.73 (15.5)	107.59 (12.55)
Faja et al., 2013	21 (16)	21 (16)	ASD	ADOS and ADI-R	82 (7.1)	80.3 (7.6)	104 (11.6)	109.1 (7.2)	107.1 (10.8)	109 (9.5)	99.1 (13)	103.4 (8)
Harris et al., 2015^*^	23 (22)	19 (NA)	Autism	ADOS and ADI-R	26	28	>85	NA	NA	NA	NA	NA
Johnson et al., 2006	15 (11)	14 (10)	AD	ADI-R	16.1 (2.3)	15.9 (2.4)	NA	NA	NA	NA	NA	NA
Lin et al., 2012	10 (7)	10 (7)	Autism or AS	ADOS and ADI-R	28 (3.1)	27 (3.1)	113 (4.7)	114 (13.4)	NA	NA	NA	NA
Mussey et al., 2015^*^	18 (NA)	15 (NA)	Autism	ADI-R	19 (2.1)	18.9 (3.3)	94.8 (27.4)	103.4 (17.2)	NA	NA	NA	NA
Schipul et al., 2012	18 (16)	18 (17)	Autism	ADOS and ADI-R	22.4 (9.6)	22.4 (4.5)	106.2 (11.9)	111.2 (6.4)	106.2 (14.9)	110.1 (6.2)	NA	NA
Solomon et al., 2015	22 (18)	25 (20)	5 HFA, 16 AS, 1 PDD-NOS (no antipsychotic medication, abstained from psychotropic medication 48 h)	ADOS-G	22.95 (5.11)	23.36 (4.15)	112.64 (12.44)	114.17 (11.51)	110.82 (13.07)	112.54 (12.32)	112.18 (12.94)	112.04 (11.53)
South et al., 2014	48 (45)	56 (42)	ASD	ADOS	13.21 (2.27)	12.57 (2.6)	109.76 (13.36)	113.82 (12.18)	NA	NA	NA	NA
Yechiam et al., 2010	15 (14)	28 (26)	12 AS, 2 PDD-NOS, 1 HFA	AQ and IC	15.6 (2.8)	15.6 (3.6)	NA	NA	NA	NA	NA	NA

*AD, Autistic Disorder; ADI-R, Autism Diagnostic Interview-Revised; ADOS, Autism Diagnostic Observation Schedule; AS, Asperger's Syndrome; ASD, Autism Spectrum Disorder; AQ, Autism Quotient; DX, Diagnosis; FSIQ-4, Full-scale Intelligence Quotient with 4 subtests; HFA, High functioning Autism; IC, independent clinicians; NA, not available; PDD-NOS, Pervasive Developmental Disorder not otherwise specified; PIQ, Perceptual Intelligence Quotient; SD, Standard Deviation; VIQ, Verbal Intelligence Quotient*.

* *Mussey et al. (2015) don't provide gender details, Harris et al. (2015) state that groups were age and sex matched but have different group sizes (Autism = 23, Control = 19)*.

In decision-making tasks with probabilistic feedback, young adults with ASD differ in their choice behavior from TD control participants. Lin et al. (2012) used an instrumental learning task during which participants had to learn the reward contingencies of three different slot machines (high-probability, low-probability, or neutral). When monetary rewards were used (win, loss, no change), individuals with ASD did not differ in their choice of the slot machine that had a high-probability of wins compared to TD individuals. However, individuals with ASD chose the high-probability slot machine significantly less if it showed social rewards (happy faces vs. angry or neutral faces) compared to TD individuals. Similarly, another study used a probabilistic selection task, in which participants had to choose between two Japanese symbols with different reward contingencies but were given correct/incorrect feedback after their choice (Solomon et al., 2015). They found that the probability of making the correct choice between two stimuli was lower in ASD participants but only when the reward contingencies were clearest (80/20 vs. 70/30 and 60/40). These authors also noted significantly lower “win-stay” choice behavior (continue choosing the same symbol after given “correct” feedback) in individuals with ASD (Solomon et al., 2015), suggesting more exploratory choice behavior. However, a relatively high number of participants with ASD were excluded from analysis due to failure to learn the task contingencies (7/17 in (Lin et al., 2012), 8/30 in Solomon et al., 2015), meaning that it is not clear how well these findings generalize across the ASD population.

The Iowa Gambling Task requires participants to choose between decks of cards to earn money, and in order to be successful, learn to avoid decks with high risk of large losses. Studies using this task have noted a tendency toward slower learning and more exploratory choice behavior in adolescents with ASD (Johnson et al., 2006; Yechiam et al., 2010; Mussey et al., 2015). However, it is notable that a study of young children (6–7 years) found that choice behavior was similar to TD peers (Faja et al., 2013) and one study of children and adolescents (8–16 years) found superior performance in high functioning ASD participants (South et al., 2014). Mixed results make interpretation difficult: while learning performance seems to be similar between children with and without ASD, studies have shown opposite results for adolescents (Johnson et al., 2006; Yechiam et al., 2010; Faja et al., 2013; Mussey et al., 2015). It would be helpful to know treatment background to understand whether superior performance can be credited to successful treatment programs employing reinforcement learning strategies.

Probabilistic reversal learning is used to assess both learning and cognitive flexibility: task contingencies change only after they are learned. Participants with ASD have shown similar initial learning, but they need a larger number of trials to acquire new contingencies after a reversal and make more regressive errors (D'Cruz et al., 2013). These differences were particularly pronounced in younger participants, suggesting a delay in the maturation of flexible behavioral control.

Further evidence for inflexible learning in ASD comes from the field of perceptual learning. It has been shown that adults with ASD show efficient initial learning, but slower learning when a target location is changed (Harris et al., 2015). This over-specificity could be reversed by reducing stimulus repetition, suggesting that protocols can be modified to circumvent inflexible learning.

While comparable brain networks are engaged during learning in both individuals with ASD and TD controls (Schipul et al., 2012), differences have been found in how these networks adapt over the course of learning (Schipul et al., 2012). One study found reduced medial prefrontal recruitment during early stages of learning a probabilistic selection task in individuals with ASD compared to TD controls and greater orbitofrontal recruitment during later stages, possibly signaling reduced transfer to working memory (Solomon et al., 2015).

### Summary

A number of interesting findings have emerged from studies of decision-making in ASD, including reduced choices for high-probability social rewards (Lin et al., 2012), more exploratory choice behavior (Solomon et al., 2015) and less flexible behavior in the context of changing contingencies (Johnson et al., 2006; Yechiam et al., 2010; Mussey et al., 2015). As differences in decision-making may be more pronounced under certain reinforcement schedules (Solomon et al., 2015), a thorough investigation of the influence of probabilistic feedback could be informative for the clinical application of RL. Furthermore, even though a more exploratory learning style in ASD could be interpreted as a general learning difficulty, studies also show that participants with ASD do show initial learning in a range of tasks such as eyeblink conditioning (Sears et al., 1994), operant learning (Salmond et al., 2003; Bernier et al., 2005; South et al., 2011), or perceptual learning (Harris et al., 2015). Learning impairments may instead reveal themselves under changing task contingencies (D'Cruz et al., 2013; Harris et al., 2015) which is in line with the fact that perseveration and inflexibility is often seen in ASD. These findings may represent a fundamental difference between fronto-sub-cortical and cerebellar learning systems; a within-subjects comparison of different learning tasks could shed more light on this question.

Studies of decision making with reward and punishment outcomes can offer a laboratory model for real-world learning situations, and when combined with neuroimaging, elucidate differences in neural mechanisms of learning. However, a gap currently exists in the literature as it is unclear whether performance on laboratory-based tasks translates to real-world learning in the context of behavioral training.

## Factors affecting response to reinforcement-based intervention

On the whole behavioral treatments appear to be beneficial, however, individuals vary in their response to these programs (Sallows and Graupner, 2005; Sherer and Schreibman, 2005; Schreibman et al., 2009; Perry et al., 2011). Several studies have sought to identify profiles or characteristics of individuals that predict improvements in IQ, adaptive behavior and social communication in response to treatment. Some, but not all (e.g., Smith et al., 2000), studies have identified pre-treatment IQ as a predictor of successful outcomes, with more impaired children showing less improvement (Ben-Itzchak and Zachor, 2007; Perry et al., 2011). Children who show greater initial social responsiveness, language skills and approach behaviors toward both adults and toys have also been shown to benefit more (Sallows and Graupner, 2005; Sherer and Schreibman, 2005; Schreibman et al., 2009). Starting intensive treatment at an earlier age has also been suggested to lead to greater gains (Harris and Handleman, 2000).

An important concept in the successful deployment of reinforcement-based intervention is reinforcer preference assessment. That is, since using the individual's own preferred stimuli as reinforcers is presumed to be most effective, systematic procedure to identify such stimuli (Kang et al., 2013), or their preferred magnitudes (Trosclair-Lasserre et al., 2008), have been developed. For example, behavioral procedures that present individuals with an array of stimuli and assess their approach/choice behaviors over multiple trials have been developed and are particularly critical for identifying appropriate reinforcers when working with young and non-verbal children. A study with TD children showed that preferred stimuli may be more effective at eliciting desired behaviors as schedule requirements during learning become more challenging (Penrod et al., 2008), thus making accurate preference assessment important for the success of learning. Interestingly, for children with ASD, the number of socially mediated reinforcers a child enjoys has been shown to predict better outcome, while the number of automatic reinforcers predicts poorer outcome (Klintwall and Eikeseth, 2012). Here, socially mediated reinforcers were defined as reinforcers that a practitioner could provide to a child (e.g., food, toys) while automatic reinforcers are those behaviors that a child can produce themselves (e.g., rocking, hand flapping). Though this was relatively small, retrospective, study, these intriguing results suggest that how an individual responds to the types of reinforcers that practitioners provide can have a bearing on that individual's learning and eventual outcome.

## Implications for treatment and questions for future research

Despite the obvious connection between RL and behavioral therapies, few studies have examined whether response to reinforcement, or learning characteristics, are predictive of treatment outcomes (Klintwall and Eikeseth, 2012). However, such work could pave the way for modifying protocols to improve outcomes for children who are “non-responders.” Indeed, variation in learning and motivation could be used to define subgroups of individuals to whom targeted interventions could be provided (Lai et al., 2013).

Despite some suggestions of aberrant response to social and non-social rewards and differences in learning in ASD, a clear and consistent physiological, behavioral, or neural marker for reduced or atypical motivation is currently lacking. Variation between studies may be due to differences in paradigms, but may also be caused by inter-individual variability, which few studies have explicitly explored. Such a marker could be useful in the context of assessing reinforcer preference (Kang et al., 2013), determining the optimal magnitude of a reinforcer (Trosclair-Lasserre et al., 2008), and predicting the likelihood of response to intervention (Klintwall and Eikeseth, 2012). For example, in those individuals with reduced or inconsistent response to rewards, greater training intensity may be necessary. Thus, this work could have important implications for treatment funding and policy. At the same time, inter-individual variability places a challenge for generalizing research findings to clinical interventions that address a wide range of individuals on the autism spectrum. Specifically, most studies have investigated individuals with high functioning autism (full-scale and verbal IQ scores ≥ 70) while intervention programs often target children with more severe forms of ASD and related cognitive disabilities. Nevertheless, the reviewed research builds a foundation for evidence-based practice and can inform which direction clinical trials have to take to further develop interventions.

Functional imaging results in ASD hint at a disconnection between the desire to obtain a reward and its hedonic effects, as has been suggested in disorders such as addiction (Robinson and Berridge, 1993), depression and schizophrenia (Rømer Thomsen et al., 2015). Indeed, given the potential similarities in abnormal learning processes across psychiatric diagnoses, this could be a useful cross-diagnostic research domain within the Research Domain Criteria (RDoC; Insel et al., 2010). In individuals with major depression, pharmacological manipulation of dopamine has been shown to improve learning (Admon et al., 2016), a strategy that could be tested in treatment-resistant individuals with ASD.

## Conclusions

The strategies harnessed by ABA-style behavioral treatment approaches are complex, and despite widespread use, our understanding of how these therapies affect behavioral and cognitive changes in ASD is limited. However, accumulating evidence points to abnormalities in the processing of reinforcers and differences in learning, and flexibly adapting, in ASD. Improvements in therapeutic efficacy may be achieved through a better understanding of functional abnormalities in learning systems in ASD. Biologically informed markers for response to rewards could help individualize treatment protocols in terms of intensity and reinforcer schedules. For individuals where there are clear differences in response to reward that impede treatment progress, pharmacological (Admon et al., 2016), or brain stimulation approaches (Reinhart et al., 2015), in combination with ABA-style therapy, could be investigated as a way of improving treatment outcomes.

## Author contributions

All authors listed have made a substantial, direct and intellectual contribution to the work, and approved it for publication.

### Conflict of interest statement

The authors declare that the research was conducted in the absence of any commercial or financial relationships that could be construed as a potential conflict of interest.
